# *In Vitro* Antifungal Activity of Hexahydropyrimidine Derivatives against the Causative Agents of Dermatomycosis

**DOI:** 10.1155/2017/1207061

**Published:** 2017-10-26

**Authors:** Francislene J. Martins, César A. Caneschi, Mônica P. Senra, Gustavo S. G. Carvalho, Adilson D. da Silva, Nádia R. B. Raposo

**Affiliations:** ^1^Núcleo de Pesquisa e Inovação em Ciências da Saúde (NUPICS), Universidade Federal de Juiz de Fora, Campus Universitário, 36036-900 Juiz de Fora, MG, Brazil; ^2^Departamento de Química, Instituto de Ciências Exatas, Universidade Federal de Juiz de Fora, Campus Universitário, 36036-900 Juiz de Fora, MG, Brazil

## Abstract

Nitrogenated heterocyclic compounds are present in both natural and synthetic drugs, and hexahydropyrimidine derivatives may prove to be efficient in treating dermatomycosis causing fungi. This study evaluated the antifungal activity of four hexahydropyrimidine derivatives against the dermatomycosis causing fungi. These derivatives were synthesized, characterized, and assessed in terms of their activity against* Trichophyton mentagrophytes*,* Microsporum canis*,* Microsporum gypseum*,* Trichophyton rubrum*,* Fusarium oxysporum*, and* Epidermophyton floccosum* between concentrations 7.8 and 1,000 *μ*g mL^−1^. Scanning electron micrographs were assessed for the active derivatives and reference drugs, and these micrographs revealed that new agents cause morphological changes in fungi. The derivatives HHP1, HHP3, and HHP4 revealed poor activity against the four fungal strains (MICs range 500–1000 *μ*g mL^−1^). Compound HHP3 was found to be the best potential antifungal agent among those tested and was the most effective among all the active derivatives that caused morphological changes in the susceptible strains.

## 1. Introduction

In a recent work our research group performed biological evaluation of hexahydropyrimidines compounds, which form an interesting type of aliphatic azaheterocycle [1, 2]. Heterocyclic compounds are of great importance in medicinal chemistry, as they are used as potential targets against several bacterial and parasitic pathogens [3, 4]. Notably, the nitrogenated heterocyclic compounds with six-membered rings are given special prominence since many synthetic or natural medicines contain this structural core. Considering the recent efforts by our research group, we obtained a series of hexahydropyrimidines, which were assessed as potential antimicrobial and antiparasitic agents [1, 2]. The efficacy and versatility of these compounds in several biological assays make them potential candidates for treating fungi that cause skin diseases.

Dermatophytes frequently colonize the skin and cause diseases in animals and humans. They affect the keratinized tissues and are spread by direct contact with infected humans (anthropophilic organisms), animals (zoophilic organisms), and soil (geophilic organisms), and they may indirectly also spread through fomites [5–7].

The risk of infections caused by dermatophytes or other fungi has increased considerably over the last decades [8, 9]. The increase in fungal infections can be attributed to the increasingly growing number of immunocompromised individuals, including organ transplant recipients, HIV/AIDS patients, patients with cancer or those undergoing chemotherapy, individuals either at an advanced age or with diabetes or cystic fibrosis, hospitalized individuals with serious illness, and patients undergoing invasive procedures [9–14].

Despite the research devoted in developing new therapeutic strategies, a limited number of drugs are available for treating these fungal infections. Only four classes of drugs that interfere with the metabolic pathways of fungi are used in clinical practice: fluoropyrimidine analogs, polyenes, azoles, and echinocandins [9–15]. Some of these drugs exhibit poor efficacy, side effects (pruritus, rash, headache, and fever with caspofungin and other echinocandins), toxicity (hepatotoxicity due to the use of griseofulvin, flucytosine, and azoles; gastrointestinal and renal toxicity due to the use of amphotericin B), drug interactions, and drug resistance, in case of inappropriate administration of the drugs [16, 17].

Considering these factors, an imperative need arises for innovative antifungal products, either synthetic or natural, which combines safety with antifungal efficacy against the most common pathogens. In the present paper, our research group explored certain synthetic hexahydropyrimidine derivative compounds, which could potentially be used for the treatment of fungal diseases through a simple and economic strategy.

Thus, this study aimed to investigate the* in vitro* antifungal activity of the hexahydropyrimidine derivatives against the filamentous fungi, which can cause dermatomycosis, for example,* Microsporum canis*,* Microsporum gypseum*,* Trichophyton mentagrophytes*,* Trichophyton rubrum*,* Fusarium oxysporum, *and* Epidermophyton floccosum*, and to observe any morphological changes in these fungi by using scanning electron microscopy (SEM).

## 2. Materials and Methods

### 2.1. Synthesis

**Scheme 1:**
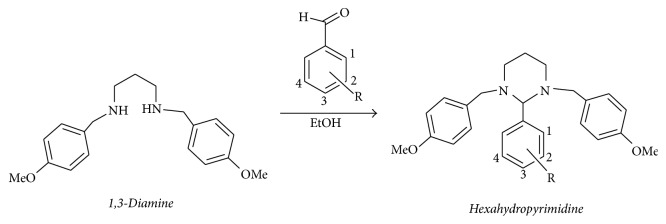
Synthetic pathway for hexahydropyrimidine derivatives adapted from De Carvalho et al. [2]. HHP1 - R_3_ = NMe_2_; HHP2 - R_1_ = OH; HHP3 - R_2_ = R_3_ = R_4_ = OMe; HHP4 - R_1_ = NO_2_.

The synthetic route is presented in Scheme 1. A detailed study of the chemical and structural elucidations of the four analogs has been described by our group in a previous article [2].

### 2.2. Fungal Strains

We tested six filamentous fungal strains:* Trichophyton mentagrophytes* ATCC 9538,* Microsporum canis* ATCC 32903,* Microsporum gypseum* ATCC 14683,* Trichophyton rubrum* CCT 5507 URM 1666,* Fusarium oxysporum* ATCC 48112, and* Epidermophyton floccosum CCF-IOC-3757*. We employed standard strains of these filamentous fungi from the Collection of Tropical Crops (CCT) provided by Foundation André Tosello, Campinas, São Paulo (Brazil) and from the* American Type Culture Collection (ATCC)* provided by the National Institute of Quality Control in Health, Oswaldo Cruz Foundation, Rio de Janeiro (Brazil).

### 2.3. Microbiological Screening

Similar to the method described by Souza et al. [18], this assay was performed for a preliminary evaluation of antifungal potential of the compounds. A 2 mm fungal fragment was placed in a culture medium of Sabouraud dextrose agar (SDA) containing hexahydropyrimidine derivatives (1,000 *μ*g mL^−1^). Later, the plates were incubated at 28 ± 2°C and were analyzed after 7 days of incubation. Endpoint was visually determined based on the growth in wells containing the drugs with that of the growth control.

### 2.4. Determination of Minimum Inhibitory Concentration (MIC)

The MIC values for the hexahydropyrimidine derivatives were evaluated according to protocols M 38-A and M 38A2 of the Clinical and Laboratory Standards Institute (CLSI) [19, 20]. This was performed only for strains revealing no visible fungal growth when exposed to 1,000 *μ*g mL^−1^ derivatives in microbiological screening.

The fungal suspension was prepared from 7-day-old cultures incubated in SDA, using 2 mL of 0.85% sterile saline and 20 *μ*L of tween-20 solution. Each suspension was diluted (1 : 50, v/v) with RPMI-1640 medium (Sigma Aldrich, USA), buffered with 3-N-morpholino propane sulfonic acid (MOPS, JT Baker, Germany), to obtain the final inoculum concentration of 0.4 to 5 × 10^4^ colony forming units (CFU). Each assay was carried out in triplicate.

Terbinafine and ketoconazole were evaluated in concentrations from 0.0234 to 480 *μ*g mL^−1^ and from 0.0313 to 640 *μ*g mL^−1^, respectively, in RPMI-1640 medium buffered with MOPS [19, 20]. Dimethyl sulfoxide analytical grade (Sigma Aldrich, USA) was used to solubilize these reference drugs [19].

The heterocycle derivatives were solubilized in the culture medium RPMI-1640 buffered with MOPS and were evaluated at eight concentrations in the range from 7.8 to 1,000 *μ*g mL^−1^.

Aliquots of 100 *μ*L of the fungal suspensions were inoculated in the microtiter plate wells (Sarstedt, Germany) containing 100 *μ*L reference drugs or analogs.

Furthermore, the microdilution plate was homogenized for 2 min and incubated at 28 ± 2°C for 7 days. The MIC was defined as the lowest concentration indicating 100% growth inhibition.

### 2.5. Minimum Fungicidal Concentration (MFC)

After evaluating MIC, the MFC was determined according to a protocol by Magagnin et al. [21]; herein, aliquots of 10 *μ*L of the medium lacking fungal growth were transferred to new 96-well microplates containing 200 *μ*L of Sabouraud dextrose broth devoid of any antifungal agent.

### 2.6. Scanning Electron Microscopy (SEM)

In order to investigate the structural alterations of filamentous fungi following different treatments, SEM was used to check the cultured fungal colonies. For this experiment, the fungal fragments were processed according to Martinelli and Santos [22] and Mio et al. [23] using the Karnovsky solution (2.5% glutaraldehyde, 2.5% formaldehyde in 0.05 M sodium cacodylate buffer, and 0.001 M calcium chloride pH = 7.2) for 24 h. This was followed by further contact with a solution of 1% osmium tetroxide for 1 h in the dark and dehydration in increasing concentrations of acetone (25%–100%). Moreover, the fragment was fixed on the aluminum stubs with the aid of a double-sided carbon tape and was attached to the metallizer (Balzers FL-9496, Liechtenstein), with a gold steam bath for 180 s. A JEOL JSM 6390 LV field emission SEM (Tokyo, Japan) was used at an acceleration voltage of 15 KV. The analyses were performed at the Scanning Electron Microscopy Laboratory of the National Museum of the Federal University of Rio de Janeiro (Rio de Janeiro, Brazil).

## 3. Results and Discussion

Screening of antifungal activity demonstrated the ability of HHP1, HHP3, and HHP4 to only inhibit the growth of the species* T. mentagrophytes* ATCC 9538,* M. canis* ATCC 32903,* M. gypseum* ATCC 14683, and* T. rubrum* CCT 5507 URM 1666, which were then subjected to the MIC and MFC tests (Table 1).

*F. oxysporum* ATCC 48112 and* E. floccosum *CCF-IOC-3757 species were not inhibited by the compounds tested at 1,000 *μ*g mL^−1^ concentrations in the microbiological screening; therefore, these species were subjected to the MIC and MFC tests. Notably, it should be noted that the reference drugs, ketoconazole and terbinafine, were unable to inhibit the fungal growth of these strains at concentrations of 640 *μ*g mL^−1^ and 480 *μ*g mL^−1^, respectively. These concentrations are extremely high, since the CLSI recommends testing ketoconazole at 16 *μ*g mL^−1^ concentration [19] and literature data reveals that both dermatophytes [24] and* F. oxysporum *[25] are sensitive to concentrations up to 32 *μ*g mL^−1^ of terbinafine. Guo and coworkers [26] found that while synthesizing a Schiff base derivative of chitosan 2-(2-hydroxyl-5-chlorobenzaldimino)-6-carboxymethyl chitosan, 43% inhibition was observed for* F. oxysporum *f. sp.* vasinfectum* at a concentration of 500 *μ*g mL^−1^, which is the most effective antifungal agent verified by the authors against this species.

Data helped confirm that the compound HHP3 was the most active among the hexahydropyrimidine derivatives tested, followed by HHP4 and HHP1, whereas HHP2 revealed no antifungal activity for such species. Compound HHP3 revealed methoxy substituents attached to positions 2, 3, and 4 of the benzene ring. According to Thanusu et al. [27], the introduction of these substituents in the* para* position of the benzene ring promoted increased antifungal activity, exhibiting the lowest MIC values against* Aspergillus flavus *and* Candida albicans*. Notably, the compound HHP3 was the most lipophilic among the products analyzed, which could facilitate its penetration into the fungal cell and thus favor its activity [28, 29]. Ouf et al. [30] synthesized triazolic derivatives and reported that when the methoxy group was present at the 4th position of the aromatic ring, the compound analyzed reduced the synthesis of ergosterol in* T. rubrum* (83.5%),* T. terrestre* (79.6%),* E. floccosum* (87.1%), and* M. gypseum* (83.3%). In addition, this compound promoted complete healing of animals (guinea pigs) infected with any of the dermatophytes.

According to Reddy et al. [31] and Shah et al. [32], the nitro or amino substituents enhance the antifungal activity, as such groups are present in compounds HHP4 and HHP1, respectively. For compound HHP4, with* nitro* substituents at the* ortho* position, it is emphasized that the movement of these substituents to the* para *position can increase the biological activity of HHP4. This finding is in correlation with other studies, suggesting that the synthesizing of a triazole derivative with coumarin moiety, in presence of the nitro group at the* para* position of the benzene ring, makes the compound's antifungal activity comparable to that of ketoconazole (12.5 *μ*g mL^−1^) [33].

The analysis of the HHP2 chemical structure showed that the pair of nitrogen electrons found itself committed to the intramolecular hydrogen bonding, which may have contributed so that it was not active under the experimental conditions. Rezaei et al. [34], while considering the imidazole and triazole compounds, stated that the nitrogen atom is essential for the interaction with the heme iron in the active site of the CYP51 enzyme, which is important for sterol biosynthesis in eukaryotes. Kokil et al. [33] emphasized that the affinity between the nitrogen atom and the apoprotein defines the antifungal selectivity. According to Shalini et al. [35], the heterocyclic compounds with nitrogen deserve special attention due to their known biological properties. Notably, Sangshetti and Shinde [36] stated that changes in the substituents linked to the nitrogen heteroatoms in the 6-membered ring might foster increased antifungal activity. In their studies, the authors found that the activity of the synthesized compounds increased when the nitrogen of piperidine was replaced with methyl, ethyl, or benzoyl substituents. They also found that when the substituent is benzoyl, the substance was equipotent to miconazole drug against the species* F. oxysporum*.

**Figure 1:**
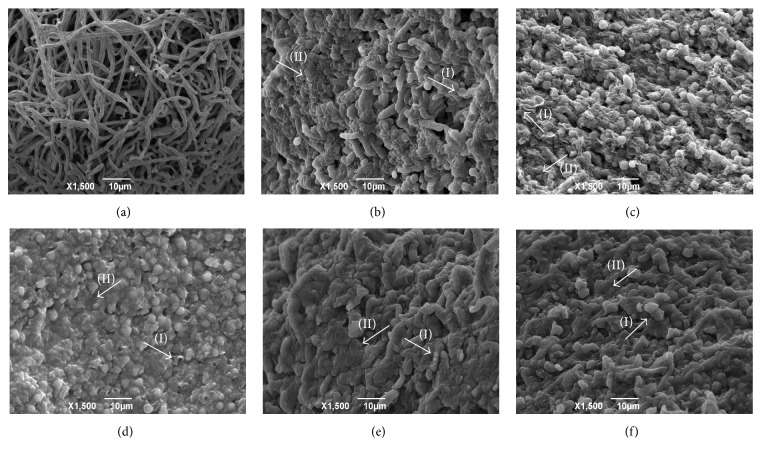
Electron micrographs of* T. mentagrophytes* ATCC 9538 exposed or not to the reference drugs and hexahydropyrimidine derivative of interest. (a)* T. mentagrophytes* ATCC 9538 not subjected to pharmacological treatment; (b)* T. mentagrophytes* ATCC 9538 subjected to treatment with terbinafine; (c)* T. mentagrophytes* ATCC 9538 subjected to treatment with ketoconazole; (d)* T. mentagrophytes* ATCC 9538 subjected to treatment with HHP1; (e)* T. mentagrophytes* ATCC 9538 subjected to treatment with HHP3; (f)* T. mentagrophytes* ATCC 9538 subjected to treatment with HHP4. Arrows indicate the appearance of wrinkled (I) and crushed (II) hyphae.

**Figure 2:**
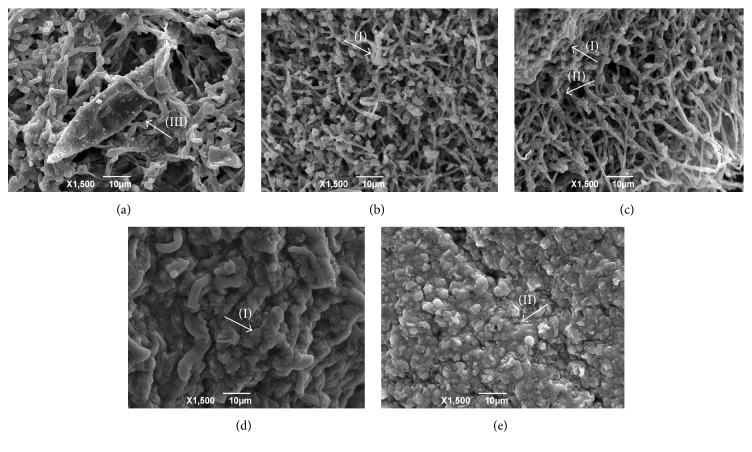
Electron micrographs of* M. canis *ATCC 32903 exposed or not to the reference drugs and hexahydropyrimidine derivative of interest. (a)* M. canis *ATCC 32903 not subjected to pharmacological treatment; (b) fungus* M. canis *ATCC 32903 subjected to treatment with terbinafine; (c)* M. canis *ATCC 32903 subjected to treatment with ketoconazole; (d)* M. canis *ATCC 32903 subjected to treatment with HHP3; (e)* M. canis *ATCC 32903 subjected to treatment with HHP4. Arrows indicate the appearance of wrinkled (I) and crushed (II) hyphae and the presence of macroconidium (III).

**Figure 3:**
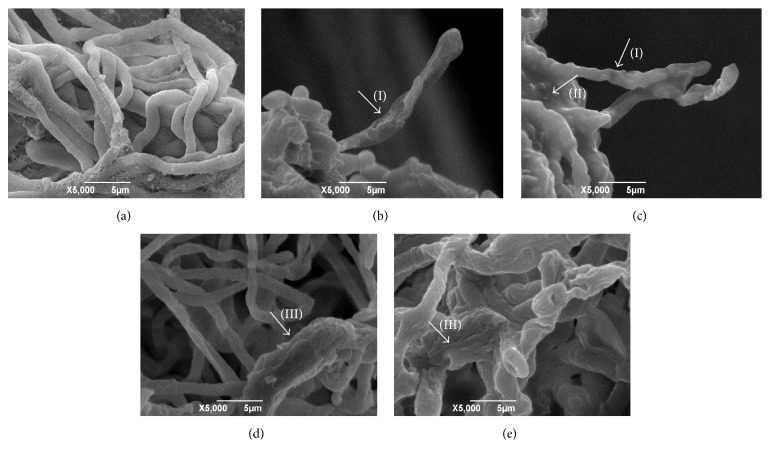
Electron micrographs of* M. gypseum *ATCC 14683 exposed or not to the reference drugs and hexahydropyrimidine derivative of interest. (a)* M. gypseum *ATCC 14683 not subjected to pharmacological treatment; (b)* M. gypseum *ATCC 14683 subjected to treatment with terbinafine; (c)* M. gypseum *ATCC 14683 subjected to treatment with ketoconazole; (d)* M. gypseum *ATCC 14683 subjected to treatment with HHP3; (e)* M. gypseum *ATCC 14683 subjected to treatment with HHP4. Arrows indicate the appearance of wrinkled (I) and crushed (II) hyphae and wrinkled macroconidium (III).

**Figure 4:**
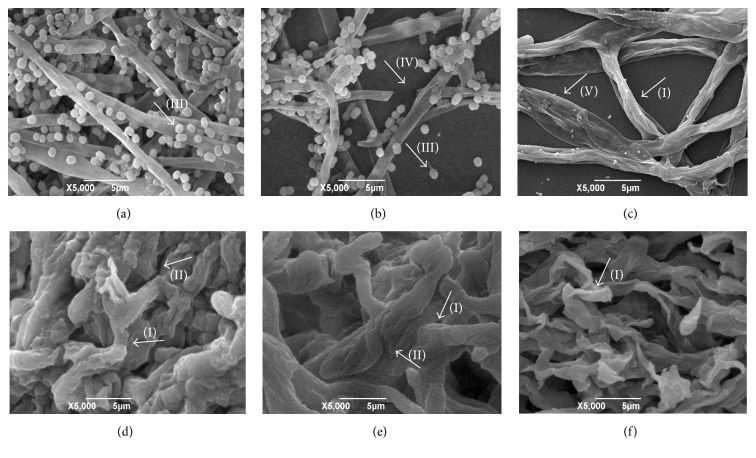
Electron micrographs of* T. rubrum *CCT 5507 URM 1666 exposed or not to the reference drugs and hexahydropyrimidine derivative of interest. (a)* T. rubrum *CCT 5507 URM 1666 not subjected to pharmacological treatment; (b)* T. rubrum *CCT 5507 URM 1666 subjected to treatment with terbinafine; (c)* T. rubrum *CCT 5507 URM 1666 subjected to treatment with ketoconazole; (d)* T. rubrum *CCT 5507 URM 1666 subjected to treatment with HHP1; (e)* T. rubrum *CCT 5507 URM 1666 subjected to treatment with HHP3; (f)* T. rubrum *CCT 5507 URM 1666 subjected to treatment with HHP4. (I) Wrinkled hyphae. (II) crushed hyphae. (III) microconidia. (IV) hyphal breaks. (V) loss of hyphae intracellular content.

The electron micrographs of fungal strains (exposed or not to the reference drugs and hexahydropyrimidine derivatives of interest) can be viewed in Figures 1–4.

Through morphological evaluation of the strains of interest by means of SEM, it was observed that the fungi* T. mentagrophytes *ATCC 9538,* M. gypseum *ATCC 14683, and* T. rubrum *CCT 5507 URM 1666 exhibited hyphae with normal and continuous growth patterns when not subjected to pharmacological treatment (Figures 1(a), 3(a), and 4(a)). Moreover, it was possible to observe the presence of numerous microconidia in* T. rubrum* CCT 5507 URM 1666 (Figure 4(a)). The fungi* T. mentagrophytes* ATCC 9538 and* M. gypseum *ATCC 14683, when treated with terbinafine and ketoconazole (Figures 1(b), 1(c), 3(b) and 3(c)), revealed changes such as hyphae with irregular, wrinkled, and crushed growth patterns. Treatment of* T. rubrum* CCT 5507 URM 1666 with terbinafine caused a reduction in the number of microconidia and the presence of the broken down hyphal regions (Figure 4(b)), while ketoconazole caused the loss of hyphae intracellular content and wrinkling (Figure 4(c)).

The* M. canis* ATCC 32903 electron micrographs revealed that the untreated fungus indicated a reproductive structure with a well-defined surface (Figure 2(a)). Macroconidia were not observed after treatment with ketoconazole (Figure 2(c)), but crushings and wrinkles were noticed in certain regions, as well as numerous hyphae. After treatment with terbinafine (Figure 2(b)), wrinkled and irregular fungal structures were observed.

After pharmacological treatment of the fungi* T. mentagrophytes *ATCC 9538 with HHP1, HHP3, and HHP4 (Figures 1(d)–1(f));* M. canis *ATCC 32903 with HHP3 and HHP4 (Figures 2(d)-2(e));* M. gypseum *ATCC 14683 with HHP3 and HHP4 (Figures 3(d)-3(e)); and* T. rubrum *CCT 5507 URM 1666 with HHP1, HHP3, and HHP4 (Figures 4(d)–4(f)), the colonies appeared compact, wrinkled with furrows, and constricted. Moreover, the crush zones were observed in these samples. In Figures 3(d)-3(e) the presence of wrinkled macroconidia was observed.

Studies have revealed the hepatotoxicity risks associated with ketoconazole [37, 38] and terbinafine [39], interference with the metabolism of other drugs [37, 40], and resistance of microorganisms to the available treatments [41, 42]. Thus, the data presented may be promising, since the structural changes can be altered in the molecules of* hexahydropyrimidine* derivatives in order to enhance their antifungal activity.

## 4. Conclusions

The* hexahydropyrimidine* derivative HHP3 was active against* T. mentagrophytes *ATCC 9538,* M. canis *ATCC 32903,* M. gypseum *ATCC 14683, and* T. rubrum *CCT 5507 URM 1666 and presented the best potential antifungal activity among the four analogs tested. None of these analogs, as well as the drugs ketoconazole and terbinafine, were active against* F. oxysporum *ATCC 48112 and* Epidermophyton floccosum *CCF-IOC-3757. The scanning electron micrographs revealed changes in the fungi structure when subjected to pharmacological treatments, which may be compatible with their mechanism of action. This study may represent the initiative for additional investigation for the role of hexahydropyrimidine derivatives molecules, as well as for development of novel active antifungal agents obtained from structural changes.

## Figures and Tables

**Table 1:** Antifungal activity of hexahydropyrimidine derivatives and reference drugs.

Compound		*T. mentagrophytes* ATCC 9538	*M. canis* ATCC 32903	*M. gypseum* ATCC 14683	*T. rubrum* CCT 5507 URM 1666
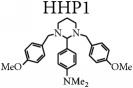	MIC MFC	1,000 1,000	>1,000 >1,000	>1,000 >1,000	500 500
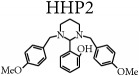	MIC MFC	>1,000 >1,000	>1,000 >1,000	>1,000 >1,000	>1,000 >1,000
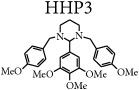	MIC MFC	500 1,000	500 1,000	500 1,000	250 500
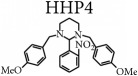	MIC MFC	1,000 >1,000	1,000 >1,000	1,000 >1,000	250 1,000
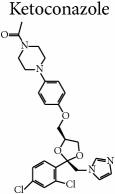	MIC MFC	1 4	8 16	10 160	16 16
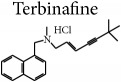	MIC MFC	3 3	0.1875 0.1875	0.1171 0.1171	0.1875 0.1875

MIC = minimal inhibitory concentration; MFC = minimal fungicidal concentration. The results were expressed in *µ*g mL^−1^.

## References

[1] De Carvalho G. S. G., Machado P. A., De Paula D. T. S., Coimbra E. S., Da Silva A. D. (2010). Synthesis, cytotoxicity, and antileishmanial activity of N,N′-disubstituted ethylenediamine and imidazolidine derivatives. *The Scientific World Journal*.

[2] De Carvalho G. S. G., Dias R. M. P., Pavan F. R. (2013). Synthesis, cytotoxicity, antibacterial and antileishmanial activities of imidazolidine and hexahydropyrimidine derivatives. *Medicinal Chemistry*.

[3] Groszkowski S., Korzycka L., Bialasiewicz W. (1973). 1,3 Di (halogenoacyl) piperimidine and 2 methylpiperimidine derivatives. *Polish Journal of Pharmacology and Pharmacy*.

[4] Horvath D. (1997). A virtual screening approach applied to the search for trypanothione reductase inhibitors. *Journal of Medicinal Chemistry*.

[5] Brown G. D., Denning D. W., Gow N. A. R., Levitz S. M., Netea M. G., White T. C. (2012). Hidden killers: human fungal infections. *Science Translational Medicine*.

[6] Nweze E. I. (2011). Dermatophytoses in domesticated animals. *Revista do Instituto de Medicina Tropical de São Paulo*.

[7] Singh T. N., Zamzachin G., Singh N. G. B. (2015). Dermatophytosis: clinico-mycological study on patients attending the department of dermatology Rims Hospital, Imphal, Manipur. *International Journal of Current Microbiology and Applied Sciences*.

[8] Jessup C. J., Warner J., Isham N., Hasan I., Ghannoum M. A. (2000). Antifungal susceptibility testing of dermatophytes: establishing a medium for inducing conidial growth and evaluation of susceptibility of clinical isolates. *Journal of Clinical Microbiology*.

[9] Vandeputte P., Ferrari S., Coste A. T. (2012). Antifungal resistance and new strategies to control fungal infections. *International Journal of Microbiology*.

[10] Romani L. (2004). Immunity to fungal infections. *Nature Reviews Immunology*.

[11] Neofytos D., Lu K., Hatfield-Seung A. (2013). Epidemiology, outcomes, and risk factors of invasive fungal infections in adult patients with acute myelogenous leukemia after induction chemotherapy. *Diagnostic Microbiology and Infectious Disease*.

[12] Al Mubarak S., Robert A. A., Baskaradoss J. K. (2013). The prevalence of oral Candida infections in periodontitis patients with type 2 diabetes mellitus. *Journal of Infection and Public Health*.

[13] Tsai P.-W., Chen Y.-T., Hsu P.-C., Lan C.-Y. (2013). Study of Candida albicans and its interactions with the host: a mini review. *BioMedicine*.

[14] Person A. K., Kontoyiannis D. P., Alexander B. D. (2011). Fungal infections in transplant and oncology patients. *Hematology/Oncology Clinics of North America*.

[15] Tillotson J., Tillotson G. S. (2015). The Regulatory Pathway for Antifungal Drugs: a US Perspective. *Clinical Infectious Diseases*.

[16] Golan D. E., Tashijian A. H., Armstrong E. J., Armstrong A. W. Farmacologia: a base fisiopatológica da farmacoterapia. 2nd ed. Rio de Janeiro: Guanabara Koogan; 2009.

[17] Girois S. B., Chapuis F., Decullier E., Revol B. G. (2006). Adverse effects of antifungal therapies in invasive fungal infections: review and meta-analysis. *European Journal of Clinical Microbiology & Infectious Diseases*.

[18] Souza L. K., Oliveira C. M., Ferri P. H. (2002). Antifungal properties of Brazilian cerrado plants. *Brazilian Journal of Microbiology*.

[19] Clinical and Laboratory Standards Institute (CLSI) Método de referência para testes de diluição em caldo para a determinação da sensibilidade a terapia antifúngica dos fungos filamentosos: norma aprovada.

[20] CLSI.

[21] Magagnin C. M., Stopiglia C. D., Vieira F. J. (2011). Perfil de suscetibilidade a antifúngicos de dermatófitos isolados de pacientes com insuficiência renal crônica. *Anais Brasileiros de Dermatologia*.

[22] Martinelli P. R. P., Santos J. M. (2010). Microscopia eletrônica de varredura de fungos Nematófagos associados a Tylenchulus semipenetrans e Pratylenchus jaehni. *Bioscience Journal*.

[23] Mio L. L., Novaes Q. S., Alves E. (2006). Metodologias de preparação de amostras de ferrugem para estudos morfológicos de urediniósporos por meio de microscopia eletrônica de varredura. *Summa Phytopathologica*.

[24] Almeida L. M., Souza E. A., Bianchin D. B., Svidzinski T. I. (2009). Resposta in vitro de fungos agentes de micoses cutâneas frente aos antifúngicos sistêmicos mais utilizados na dermatologia. *Anais Brasileiros de Dermatologia*.

[25] Alastruey-Izquierdo A., Cuenca-Estrella M., Monzón A., Mellado E., Rodríguez-Tudela J. L. (2008). Antifungal susceptibility profile of clinical Fusarium spp. isolates identified by molecular methods. *Journal of Antimicrobial Chemotherapy*.

[26] Guo Z., Ren J., Dong F., Wang G., Li P. (2013). Comparative study of the influence of active groups of chitosan derivatives on antifungal activity. *Journal of Applied Polymer Science*.

[27] Thanusu J., Kanagarajan V., Gopalakrishnan M. (2010). Synthesis, spectral analysis and in vitro microbiological evaluation of 3-(3-alkyl-2,6-diarylpiperin-4-ylidene)-2-thioxoimidazolidin-4-ones as a new class of antibacterial and antifungal agents. *Bioorganic and Medicinal Chemistry Letters*.

[28] Khan N., Shreaz S., Bhatia R. (2012). Anticandidal activity of curcumin and methyl cinnamaldehyde. *Fitoterapia*.

[29] Shreaz S., Sheikh R. A., Bhatia R. (2011). Antifungal activity of *α*-methyl trans cinnamaldehyde, its ligand and metal complexes: Promising growth and ergosterol inhibitors. *BioMetals*.

[30] Ouf S. A., Taleb A. M. A., Tharwat N. A., Geweely N. S. (2013). Efficacy of some synthesized thiazoles against dermatophytes. *Journal de Mycologie Medicale*.

[31] Reddy L. M., Reddy G. D., Padmaja A., Padmavathi V. (2015). Synthesis and antimicrobial activity of amino linked heterocycles. *Chemical and Pharmaceutical Bulletin*.

[32] Shah J. J., Khedkar V., Coutinho E. C., Mohanraj K. (2015). Design, synthesis and evaluation of benzotriazole derivatives as novel antifungal agents. *Bioorganic and Medicinal Chemistry Letters*.

[33] Kokil G. R., Rewatkar P. V., Gosain S. (2010). Synthesis and in vitro evaluation of novel 1, 2, 4-triazole derivatives as antifungal agents. *Letters in Drug Design and Discovery*.

[34] Rezaei Z., Khabnadideh S., Pakshir K., Hossaini Z., Amiri F., Assadpour E. (2009). Design, synthesis, and antifungal activity of triazole and benzotriazole derivatives. *European Journal of Medicinal Chemistry*.

[35] Shalini K., Kumar N., Drabu S., Sharma P. K. (2011). Advances in synthetic approach to and antifungal activity of triazoles. *Beilstein Journal of Organic Chemistry*.

[36] Sangshetti J. N., Shinde D. B. (2010). One pot synthesis and SAR of some novel 3-substituted 5,6-diphenyl-1,2,4-triazines as antifungal agents. *Bioorganic and Medicinal Chemistry Letters*.

[37] Gupta A. K., Daigle D., Foley K. A. (2015). Drug safety assessment of oral formulations of ketoconazole. *Expert Opinion on Drug Safety*.

[38] Yan J. Y., Nie X. L., Tao Q. M., Zhan S. Y., Zhang Y. (2013). Ketoconazole associated hepatotoxicity: a systematic review and meta- analysis. *Biomedical and Environmental Sciences*.

[39] Yan J., Wang X., Chen S. (2014). Systematic review of severe acute liver injury caused by terbinafine. *International Journal of Clinical Pharmacy*.

[40] Saarikoski T., Saari T. I., Hagelberg N. M. (2015). Effects of terbinafine and itraconazole on the pharmacokinetics of orally administered tramadol. *European Journal of Clinical Pharmacology*.

[41] Sun X., Wang K., Yu X. (2014). Transcription factor CCG-8 as a new regulator in the adaptation to antifungal azole stress. *Antimicrobial Agents and Chemotherapy*.

[42] Tanriverdi S. T., Özer Ö. (2013). Novel topical formulations of Terbinafine-HCl for treatment of onychomycosis. *European Journal of Pharmaceutical Sciences*.

